# Dielectric relaxation and pinning phenomenon of (Sr,Pb)TiO_3_ ceramics for dielectric tunable device application

**DOI:** 10.1038/srep31960

**Published:** 2016-09-15

**Authors:** Xian-Xiong Huang, Tian-Fu Zhang, Xin-Gui Tang, Yan-Ping Jiang, Qiu-Xiang Liu, Zu-Yong Feng, Qi-Fa Zhou

**Affiliations:** 1 School of Physics & Optoelectric Engineering, Guangdong University of Technology, Guangzhou Higher Education Mega Center, Guangzhou 510006, People’s Republic of China; 2 NIH Transducer Resource Center and Department of Biomedical Engineering, University of Southern California, Los Angeles 90015, USA

## Abstract

The behavior of ferroelectric domain under applied electric field is very sensitive to point defects, which can lead to high temperature dielectric relaxation behaviors. In this work, the phases, dielectric properties and ferroelectric switching behavior of strontium lead titanate ceramics were investigated. The structural characterization is confirmed by X-ray diffraction. The high dielectric tunability and high figure of merit of ceramics, especially Sr_0.7_Pb_0.3_TiO_3_ (SPT), imply that SPT ceramics are promising materials for tunable capacitor applications. Oxygen vacancies induced dielectric relaxation phenomenon is observed. Pinched shape hysteresis loops appeared in low temperature, low electric field or high frequency, whereas these pinched hysteresis loops also can become normal by rising temperature, enhancing electric field or lowering frequency. The pinning and depinning effect can be ascribed to the interaction between oxygen vacancies and domain switching. A qualitative model and a quantitative model are used to explain this phenomenon. Besides, polarization and oxygen treated experiment can exert an enormous influence on pinning effect and the machanisms are also discussed in this work.

Materials with high dielectric constant and high nonlinear dependence of the dielectric constant on applied voltage are promising candidates for high tunable dielectric devices. Ferroelectric materials are very exciting class of microwave materials because their dielectric constants depend nonlinearly on an applied electric field. Ferroelectricity is one of the most used and studied phenomena in both scientific and industrial communities. Properties of ferroelectrics make them particularly suitable for a wide range of applications, ranging from sensors and actuators to optical or memory devices^1^,^2^. Those important properties are largely controlled by ferroelectric domains. Thus understanding the behavior of ferroelectric domains has been a central issue for ferroelectric research. The ferroelectric domain switching is very sensitive to point defects, which can lead to a pinched shape hysteresis loops. Many ferroelectrics show pinning effect, such as Mn-doped Pb(Zr,Ti)O_3_^3^,^4^,^5^,^6^, (Ba,Sr)TiO_3_^7^, BaTiO_3_^8^,^9^, (Pb,Sr)TiO_3_^10^, Cu-doped K_0.5_Na_0.5_NbO_3_^11^, K-doped Pb(Zr,Ti)O_3_ ceramics^12^, and BiFeO_3_ thin films^13^. Various mechanisms have previously been proposed to explain pinning effect. Previous work showed that pining effect in Pb(Mn_1/3_Nb_2/3_)O_3_-Pb(Ti,Zr)O_3_ ceramics was caused by defect dipoles, and depinning effect was detarmined by the reorientation of dipoles in high temperature region, author believed that defect dipoles resulted in pinning effect, while the depinning effect resulted from strong p-type conductivity^3^. Pandey *et al*. attributed the pinning effect to internal stress or internal field which interrupted long range polar order^14^. Besides, according to the first-principles study, Chandrasekaran *et al*. provided several atomistic simulated diagrams to explain pinning effect^15^.

(Sr,Pb)TiO_3_ is a promising material in the applications of dielectric tunability and pyroelectricity^16^,^17^,^18^,^19^. Recently, Liu *et al*. reported that a large electro-shape-memory effect (up to 0.23% under the electric field of 5 kV/mm) in Mn-doped (Pb,Sr)TiO_3_ ceramics^10^. SrTiO_3_ is a quantum paraelectric and shows dielectric relaxation at high temperatures due to oxygen vacancies^20^,^21^,^22^,^23^. While in this work, both dielectric relaxations and pinning/depinning phenomenons were observed in (Sr,Pb)TiO_3_ ceramics. Results showed that the high temperature dielectric relaxation was related to oxygen vacancies. The pinning effect could be explained by a qualitative model (the symmetry-conforming property of point defects), while the depinning effect could be explained by a quantitative model (domain wall velocity during ferroelectric switching). And, both models were different from previous researches. Moreover, the effect of polarization, O_2_ treatment and N_2_ treatment on pinning effect were also discussed.

## Two Models

It is well known that after aging, ferroelectrics could exhibit double-hysteresis-like loops^7^,^8^,^9^,^10^,^11^. Aging is a process involving gradual stabilization of ferroelectric domain by defect dipoles. A various of domain stabilization theories such as the grain-boundary theory, surface-layer model, domain-wall theory, and volume theory have been proposed^24^. Recently, based on the symmetry-conforming property of point defects (defect symmetry principle)^8^,^9^, it has been shown that the domain stabilization is a volume effect. According to this view point, point defects in crystal possess a “statistical symmetry” which follows the crystal symmetry in equilibrium ferroelectric state (i.e. aged ferroelectric state) and it is the symmetry property of point defects that creates a restoring force to drive domains back to original state during polarization switching^7^,^8^,^9^. Besides, in equilibrium state, the polar crystal symmetry can lead to a polar defect short range ordering distribution which creates a defect polarization *P*_D_ aligning along the spontaneous polarization *P*_S_ direction.

**Figure 1:**
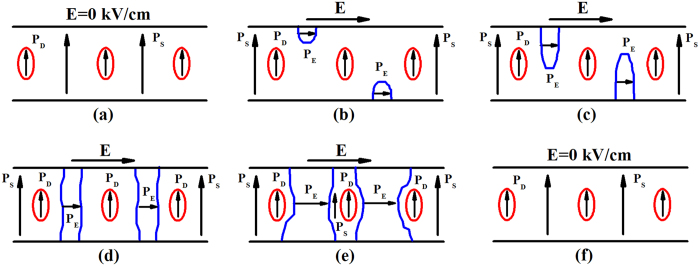
The simulated diagram of domain switching behavior of a single-domain crystal in equilibrium ferroelectric state during applied electric field cycling. Small ellipses represent defect dipoles, blue lines represent new domain walls. *P*_S_ refers to spontaneous polarization, *P*_D_ defect polarization aligning along *P*_S_, *P*_E_ polarization caused by applied electric field.

Normally, the process of polarization switching comprises four courses: (i) nucleation of the new domains; (ii) lengthwise growth of the domains; (iii) transverse growth of the domains; (iv) combination of the domains. However, it is highly probable that defect dipoles can prohibit the transverse growth of domains accord to defect symmetry principle. Figure 1 shows a series of domain patterns during electric field cycling in a single-domain crystal with defect polarization *P*_D_ aligning along the spontaneous polarization *P*_S_ direction, which is not parallel to the direction of applied electric field *E*. The sample keeps a single-domain state before applied an electric field [Fig. 1(a)]. When applied a horizontal electric field, the single-domain crystal will undergo the following several stages: (i) nucleation of the new domains [Fig. 1(b)]; (ii) lengthwise growth of the new domains [Fig. 1(c)]; (iii) transverse growth of the new domains [Fig. 1(d,e)]; (iv) back to original single-domain pattern when the electric field decreases to 0 kV/cm [Fig. 1(f)]. During transverse growth, the polar defects can hinder the domain switching and the area of original domains around *P*_D_ may be so small that it cannot be observed with a domain-level optical microscope. Thus the single-domain crystal with polar defects keeps multidomain state during electric field cycling. According to defect symmetry principle, polar defects can create a restoring force in favour of domain switching back to original single-domain pattern after removing the electric field. This process of polarization switching with point defects is different from what we have known about normal polarization switching. Besides, in order to hinder transverse growth of new domains, the point defects maybe deform the domain walls when they close to the point defects during polarization switching, and this is similar to the plastic deformation in metal where the second phase particles can deform dislocation lines when they pass the second phase particles^25^. The side-wise 180° domain wall velocity *ν* in the low-field region can be expressed by^26^:


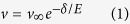


where *E* is the applied electric field, and *δ* and *ν*_∞_ are essentially field independent over the measured rang. Besides, *δ* is found to increase slightly with field and varies with temperature faster than *T*^−1^.

In the case of volume effects, another relation for the wall velocity ν is proposed^24^,


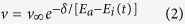


where *E*_*a*_ is the applied electric, *E*_*i*_ is the internal bias field and *δ* and *ν*_∞_ are essentially field independent over the measured rang. *δ* increases slightly with field and varies with temperature faster than *T*^−1^.

### Experimental Procedure

Strontium lead titanate ceramics (Sr_1-*x*_Pb_*x*_TiO_3_ with *x* = 0.2, 0.25, 0.3, 0.35, 0.4, 0.45, 0.5, 0.55, abbreviated as SPT100*x*) were prepared by a conventional solid-state reaction technique^19^. Reagent-grade Pb_3_O_4_, SrCO_3_ and TiO_2_ powders were weighted according to their stoichiometric composition. Then powders in stoichiometric ratio were first mixed and calcined at 850 °C for 5 h. The calcined powders were mixed with alcohol milling for 24 h and dried. After that, they were mixed thoroughly with a polyvinyl alcohol (PVA) binder solution and uniaxially pressed into discs of 12 mm in diameter and 2 mm in thickness. These discs were sintered at 1300~1350 °C for 2 h in air. Silver paste was applied on both surfaces of the discs and fired at 650 °C as electrode for electrical properties measurement.

The dielectric tunability was measured by using a blocking circuit, a multi-frequency LCR meter (Model SR720 of Stanford Research System), and a dc power source (Keithley 6517A). The permittivity and dielectric loss were measured by Agilent E4980A in the frequency range from 500 Hz to 1 MHz and in the temperature range from 25 to 550 °C. Ferroelectric hysteresis loops were obtained by a computer-controlled virtual-ground circuit with Radiant Technologies Precision Premier II(Radiant Tech, USA). Due to the retention and relaxation, the loops are usually not closed. The temperature (*T*), frequency (*ƒ*) and electric field (*E*) used here varied from 25 to 150 °C, 0.05 to 50 Hz and 10 to 40 kV/cm, respectively.

## Results and Discussion

**Figure 2:**
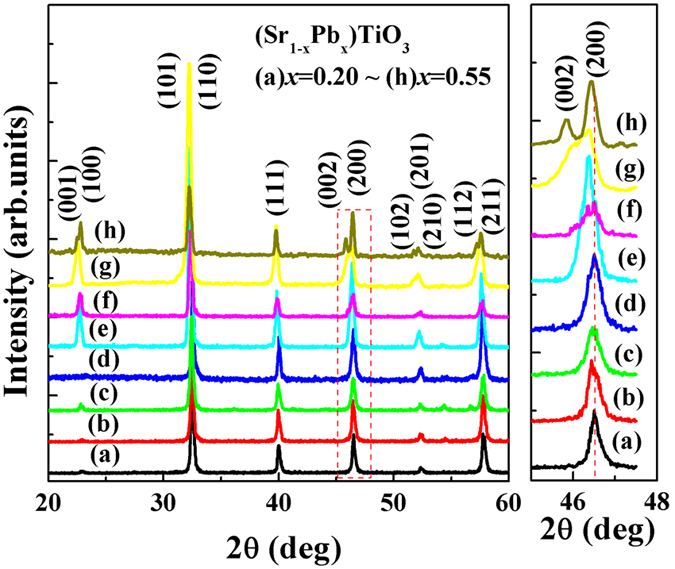
XRD patterns for SPT ceramics.

Figure 2 shows the room temperature XRD patterns of SPT ceramics with different Pb content. The XRD patterns show a typical polycrystalline perovskite structure in agreement with the PDF card No. 52–1119 and with no evidence of the secondary phase formation for all the ceramics, which indicates the Pb^2+^ ions are incorporated into the SrTiO_3_ matrix. The XRD patterns, dielectric and ferroelectric properties show a domination of a tetragonal structure. The precise peak splitting of the doublet near 45° to 48° of 2θ is included in Fig. 2. It is clearly seen that with the increase of Pb concentration, tetragonal phase in SPT ceramics becomes more and more obvious.

**Figure 3:**
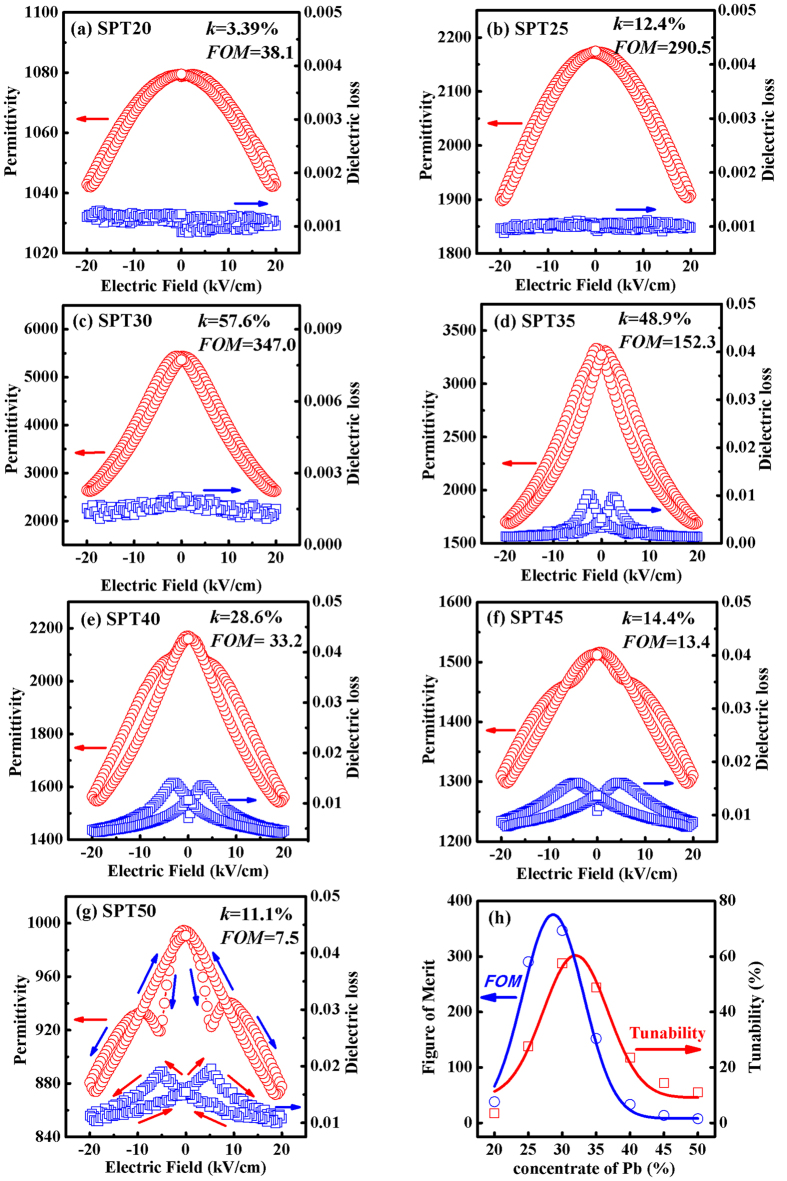
Permittivity and dielectric loss as a function of electric field for SPT20 (**a**), SPT25 (**b**), SPT30 (**c**), SPT35 (**d**), SPT40 (**e**), SPT45 (**f**) and SPT50 (**g**), which were measured at room temperature with 20 kV/cm and 10 kHz. The dielectric tunability and *FOM* are shown in (**h**).

Tunability (*k*) is defined as the ratio of the dielectric permittivity of the material at zero electric field to its permittivity at an applied electric field. Clearly, tunability is usually used to signify the strength of dielectric nonlinearity. Generally speaking, dielectric nonlinearity contains two macroscopic manifestation: ferroelectric hysteresis loops and bias characteristics of dielectric constant (tunability). So, both tunability part and hysteresis part can be used to describe the dielectric nonlinearity. Ferroelectric materials with a high dielectric tunability and high figure of merit have potential usefulness for tunable capacitors^27^,^28^. Lots of works of dielectric tunability effect have been done and have made great progress. Sun *et al*. analyzed the dielectric tunability effect in bulk LuFe_2_O_4_, they achieved dielectric tunability ahout 80% under the electric field of 50 V/cm^29^. In this paper, the dc field dependence of permittivity and dielectric loss for SPT samples at room temperature were measured under a dc field of 20 kV/cm and a frequency of 10 kHz. The results are shown in Fig. 3. However, the dielectric tunability of SPT55 is not shown here because of the strong ferroelectricity and high dielectric loss at room temperature.

The tunability (*k*) can be calculated by


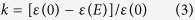


where *ε*(0) and *ε*(*E*) represent the dielectric constant at zero and a non-zero electric field, respectively. The tunabilities for SPT20, SPT25, SPT30, SPT35, SPT40, SPT45 and SPT55 are 3.39%, 12.4%, 57.6%, 48.9%, 28.6%, 14.4% and 11.1%, respectively. It can be seen that the dielectric tunability of SPT30 is higher than the others. The reason for a higher tunability is that the investigated temperature (room temperature) is near to the Curie temperature (*T*_*c*_ = 12 °C) of SPT30.

The figure of merit (*FOM*) is calculated by


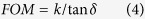


The maximum *FOM* is 347 and the corresponding sample is SPT30. Although our results of tunability (*k*) is lower than that of bulk LuFe_2_O_4_ (tunability ahout 80%), but compared to the similiar ferroelectric materials, these *k* and *FOM* of the PST, especially SPT30, are comparable to the currently studied tunable materials, such as (Ba,Sr)TiO_3_ and Ba(Zr,Ti)O_3_^27^,^28^. The tunability effect and figure of merit of the SPT ceramics, also indicate that SPT is a promising material for tunable capacitor applications.

**Figure 4:**
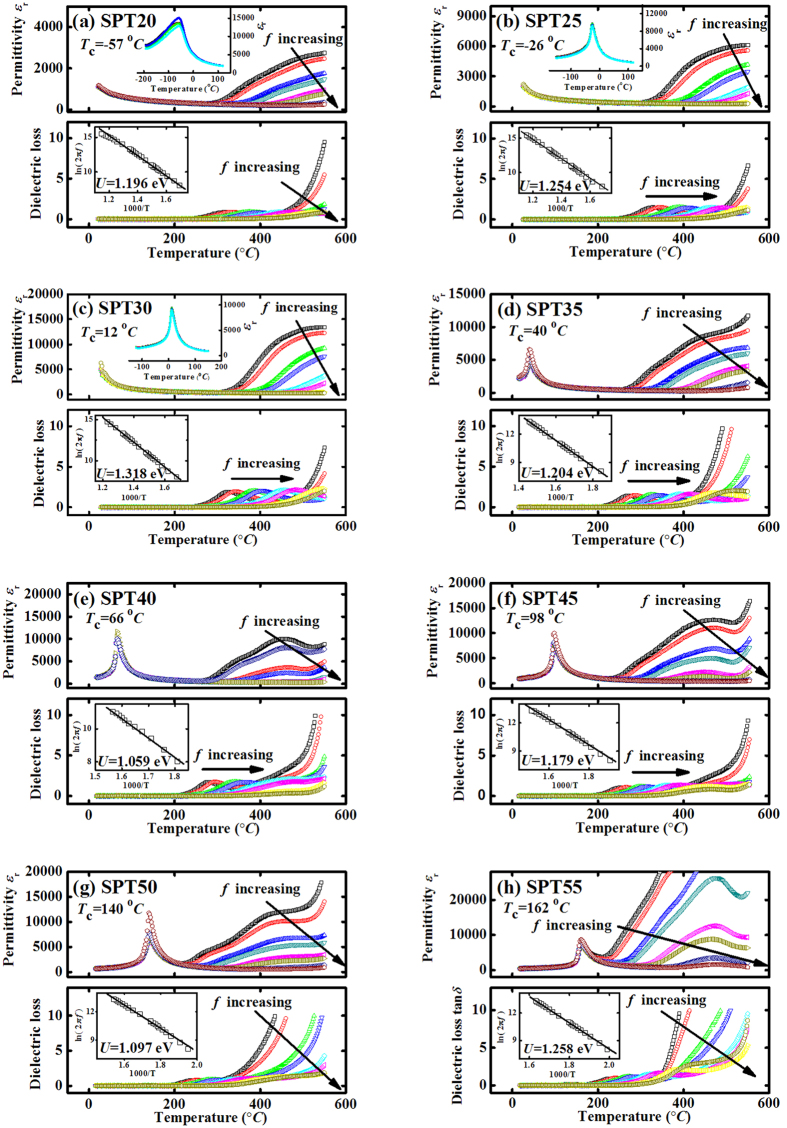
Temperature dependence of permittivity *ε*_r_ and dielectric loss tan*δ* at various frequencies of 0.5, 1, 5, 10, 50, 100, 500, and 1000 kHz for (Sr,Pb)TiO_3_ ceramics (**a**) SPT20, (**b**) SPT25, (**c**) SPT30, (**d**) SPT35, (**e**) SPT40, (**f**) SPT45, (**g**) SPT50 and (**h**) SPT55. The insets in (**a**–**c**) are the temperature dependence of permittivity between −200 ~150 °C. The insets in (**a**–**h**) give the plot between ln(2*πf*) and 1000/*T* according to the Arrhenius law.

In perovskites, the high-temperature relaxation behavior mainly occurs in lead-based compositions^28^,^30^,^31^,^32^. Figure 4 depicts the temperature dependence of permittivity (*ε*_*r*_) and dielectric loss (tan*δ*) at different frequencies. In these results, a series of wide and prominent relaxation peaks are observed. These peaks obviously shift toward a higher temperature with increasing measuring frequency, which are typical dielectric relaxation behavior. The activation energy (*U*) of relaxation units can be calculated by the famous Arrhenius law. The relaxation time (*τ*) can be written as:


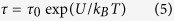


where *T* is the peak temperature of tan*δ, τ*_0_ is the relaxation time at an infinite temperature, and *k*_*B*_ is Boltzmann constant. Using the extreme value condition:


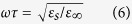


where *ε*_s_ is the static permittivity, *ε*_∞_ is the permittivity at high frequency, and angular frequency *ω* = 2*πf*, the relationship between the measuring frequency and tan*δ* peak temperature can be expressed as


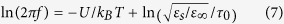


According to Eq. (5), the peak temperature of tan*δ* at different frequencies for (Sr,Pb)TiO_3_ ceramics are well fitted as shown in the inset of Fig. 4. The slopes of the linear fits yield the activation energy (*U*) and the values of activation energy are between 1.059 and 1.318 eV, which are close to the typical value (1.0 eV) of activation energy for oxygen vacancies in the perovskites^32^,^33^,^34^. So the relaxation peaks may be associated with the oxygen vacancies.

**Figure 5:**
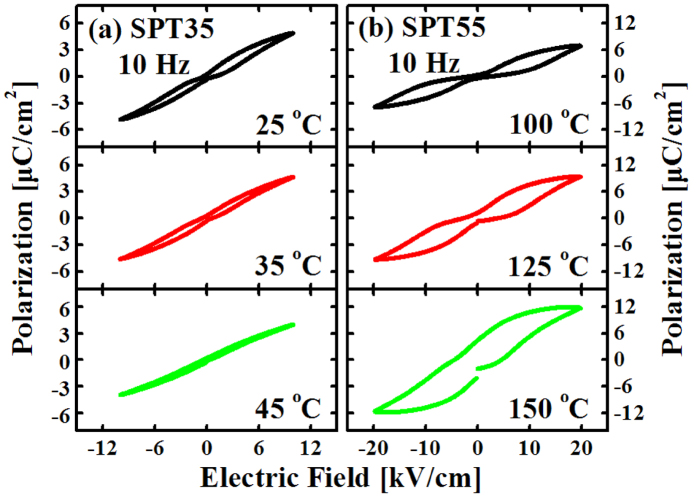
Hysteresis loops for SPT35 (**a**) and SPT55 (**b**) measured at 10 Hz and different temperatures.

The variation of *P*-*E* hysteresis loops for SPT35 and SPT55 ceramics with different temperatures is shown in Fig. 5. It can be seen that the samples exhibit pinched loops and these loops are constricted at *E*=0 kV/cm. However, as the temperature increases, these loops “open” gradually and become normal loops at 45 °C for SPT35 and 150 °C for SPT55, respectively. It is also noted that the loops of SPT35 are much thinner than that of SPT55. The reason may be that the concentration of PbTiO_3_ (ferroelectric phase) in SPT35 is much lower than that of SPT55. Moreover, on increasing the Pb/Sr ratio, the Curie temperature *T*_C_ increases gradually^10^,^16^,^19^. So the ferroelectric phase of SPT35 is much smaller than that of SPT55 at the same temperature, and this case may also result in a thin loop.

**Figure 6:**
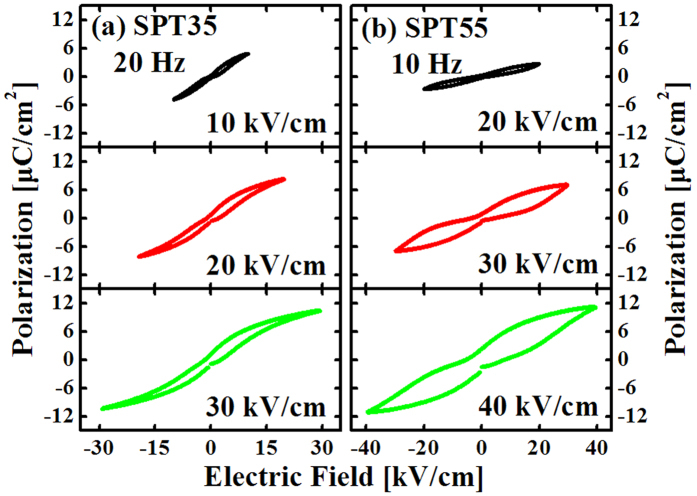
Hysteresis loops for SPT35 (**a**) and SPT55 (**b**) measured at 10 Hz, room temperature and different electric fields.

**Figure 7:**
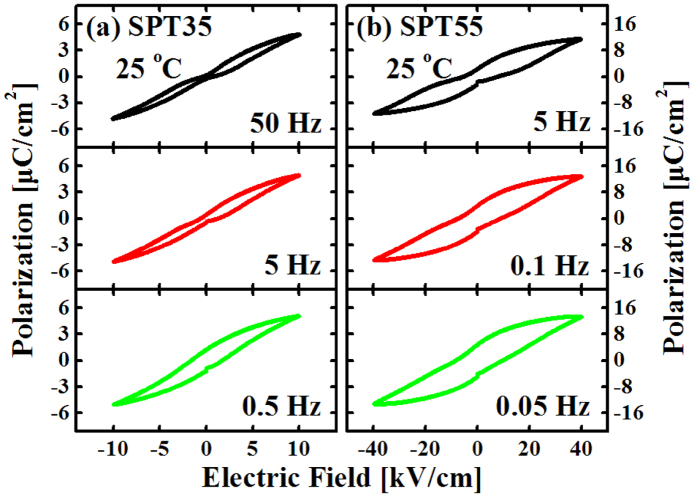
Hysteresis loops for SPT35 (**a**) and SPT55 (**b**) measured at room temperature and different frequencies.

Figures 6 and 7 show the electric field and frequency dependent hysteresis loops for SPT35 and SPT55 ceramics, respectively. The effect of enhancing electric field or lowing frequency on the loops has a tendency similar to that of rising temperature. The loops become “swelled” gradually with increasing electric field or lowing frequency and turn into normal loops finally.

**Figure 8:**
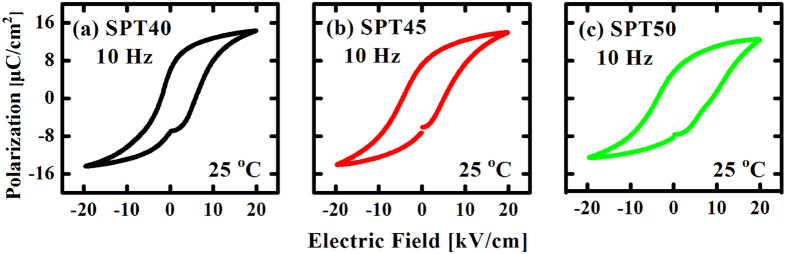
Hysteresis loops for SPT40 (**a**), SPT45 (**b**) and SPT50 (**c**) measured at room temperature.

Figure 8 shows the hysteresis loops for SPT40, SPT45 and SPT50 ceramics. As we can see, these loops are normal loops and do not show pinning effect. The reason may be that samples do not produce enough oxygen vacancies to pin the domain switching. More detail will be discussed in the following. Curie temperatures of SPT20, SPT25 and SPT30 are lower than room temperature, so their ferroelectric properties were not discuss in this work.

Just as mentioned previously, the acceptor-doped ferroelectric in aging state exhibits a double-hysteresis-like loop and the existence of defect dipoles (acceptor ions and oxygen vacancies) is the primary cause of pinning effect. Recently, the research about oxygen vacancies in materials has been done from theory and experiment^4^,^5^,^12^,^15^,^21^,^35^,^36^,^37^,^38^,^39^,^40^,^41^,^42^. Li *et al*. and Zhang *et al*. analyzed the photoemission spectra of O 1s by X-ray photoelectron spectrum (XPS) and provided a direct evidence for the existence of oxygen vacancies in acceptor-doped ferroelectrics^4^,^5^. According to defect symmetry principle, oxygen vacancies and aging is the key factor of pinning effect. In this paper, the undoped (Sr,Pb)TiO_3_ ceramics with pinched shape loops are known to contain oxygen vacancies and lead vacancies, which are created by the evaporation of lead oxide during sintering process at high temperatures. In (Pb,Zr)TiO_3_, previous researchers also observed the pinning effect which has a close relationship with oxygen vacancies^4^,^6^. Before measured, ceramics were kept at high temperature environment. The time is so long that the specimens become actually aged ferroelectrics. So the defect symmetry principle can be applied to (Sr,Pb)TiO_3_ ceramics. Due to easy formation of oxygen vacancies and lead vacancies in lead-based perovskites, it is likely that the oxygen-lead divacancy could exist in an undoped material and form defect dipoles^15^. When an external electric field is applied to the sample, domain switching occurs abruptly (without diffusion). Based on previous research results^12^,^37^, the defect polarization *P*_D_ cannot be rotated in such a diffusionless domain switching process, since the reorientation of *P*_D_ involves the oxygen vacancies migration and requires long time, thermal energy and high electric field, *i.e.*, more energy^38^. The unchanged *P*_D_ provides a restoring force favouring the domains back to original state when the electric field is removed, so a pinched *P*-*E* loop is observed. From the experimental results, SPT40, SPT45 and SPT50 do not show pinched loops. The reason may be that the oxygen vacancies produced by lead oxide evaporation during sintering is so less that available defect dipoles are less and cannot effectively hinder domain switching. So the loops observed are normal. In addition, it is interesting to see that the activation energies of oxygen vacancies in SPT35 (1.204 eV) and SPT55 (1.258 eV) with pinning effect are higher than that in SPT40 (1.059 eV), SPT45 (1.179 eV) and SPT50 (1.097 eV) with no pinning effect.

Concerning the depinning effect, previous researchers consider the migration of some oxygen vacancies along the direction of applied electric field at high temperature or low frequency as the main cause^9^,^11^. However, in this paper, the same pinched loops can be observed immediately again at low temperature, low electric field or high frequency after the sample is measured at high temperature, high electric field or low frequency. If the pinning phenomenon can be observed again after depinning, it should involve the migration of oxygen vacancies so that the defect dipoles can pin domain switching. However, as discussed above, the oxygen vacancies migration is difficult without sufficient energy or long time. So the oxygen vacancies migration may not be the main cause for depinning effect and there should be another explanation for the observed phenomenon.

According to Eq. (1) or Eq. (2), as temperature or electric field raises, the domain wall velocity *ν* increases. In other words, new domain wall can move fast toward defect dipoles at high temperature or high electric field. As a result, the area of original domain around defect dipoles is small and the restoring force is insufficient to reverse the domain to original state when the external electric field decreases to zero. Thus the loops become normal. While in the same velocity condition, the domain walls have enough time to close to defect dipoles in low-frequency measurement and the area of original domain around defect dipoles is also small. The reversal of domain to original state cannot be completed when the field decreases to zero, thus resulting in depinning effect. Besides, electrical conduction in samples at high-temperature, high-electric-field or low-frequency measurements can also contribute to the depinning effect.

**Figure 9:**
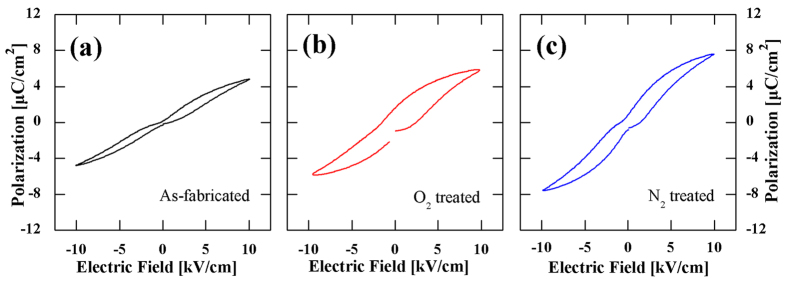
Hysteresis loops for SPT35 measured at 50 Hz, 10 kV/cm and room temperature, (**a**) as fabricated, (**b**) O_2_ treated and (**c**) N_2_ treated.

To clarify the origin of pinning effect, a special treatment has been done for SPT35 sample. After measurements of hysteresis loops in different situations, the same sample was annealed in O_2_ atmosphere at 600 °C for 10 h. Then a slightly constricted hysteresis loop was observed, as shown in Fig. 9. Annealing in oxygen can reduce oxygen vacancies^21^. When the concentration of oxygen vacancies is low, the restoring force from oxygen vacancies is insufficient to reverse the domain to original state, resulting in a slightly contractive loop. This result indicates that the oxygen vacancies are the main cause for pinning effect in undoped (Sr,Pb)TiO_3_ ceramics and their concentration can affect the extent of pinning. It is consistent with the discussion about normal *P*-*E* loops in SPT40, SPT45 and SPT50. After the measurements of O_2_ treated SPT35 sample, SPT35 sample was then annealed in N_2_ atmosphere at 600 °C for 10 h. Then typical constricted hysteresis loop was recovered, as shown in Fig. 9c. This strongly indicates that oxygen vacancies and its concentration plays an important role in pinning effect. Additional, it could be seen that values of polarization increased slightly after N_2_ treatment, which might be contributed by some defects (oxygen vacancies).

**Figure 10:**
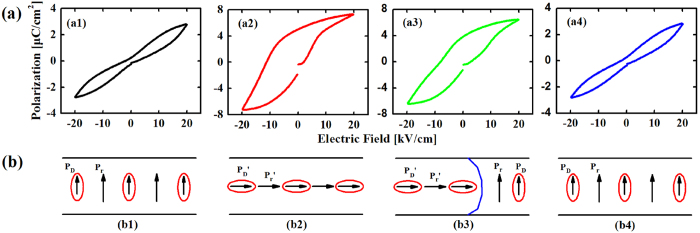
Hysteresis loops (**a**) and simulated diagrams of domain switching behavior (**b**) for SPT55 which measured before being poled (a1) and (b1), immediately after being poled (a2) and (b2), one day later after being poled (a3) and (b3) and after annealing in air (a4) and (b4). *P*_S_, *P*_S_’, *P*_D_ and *P*_D_’ represent spontaneous polarization, new spontaneous polarization after poled, defect polarization and new defect polarization after poled, respectively. The blue line in (b3) is domain wall.

An additional work has been done for SPT55, which is selected and poled at 160 °C, 15 kV/cm for 30 min. After poled, *P*-*E* loops of SPT55 were measured. The result is showed in Fig. 10. It was interesting to see that the sample did not exhibit pinched loop which was measured immediately after poled. While a slightly constricted loop was observed after 24 hours and a severely constricted loop was observed after annealing in air. This phenomenon can be explained as follow. Oxygen vacancies may obtain enough energy to migrate and form new defect polarization *P*_D_’ parallel with the direction of applied electric field during the sample being poled. The parallel defect polarization *P*_D_’ creates less force for reversible domain switching, so a loop with no pinning effect is observed. Over time, a part of single domain will change the orientation of polarization due to the influence of surrounding environment *e.g*. thermal motion, so will defect dipoles. These defect dipoles unparallel to applied electric field can hinder the transverse growth of new domain and afford restoring force for reversible domain switching, resulting in a lightly constricted loop. After annealing, the polarization state in sample can return to original state. Consequently, a severely constricted loop is observed. When sample is poled, one direction is easy to reach saturated polarization and the other is difficult to reach saturated polarization, as shown in Fig. 9(a2,a3).

## Conclusions

In this work, a detailed study on the structures, dielectric properties and ferroelectric domain switching of (Sr,Pb)TiO_3_ ceramics is investigated. The XRD pattern shows a domination of a tetragonal structure in SPT ceramics. The high dielectric tunability and high figure of merit imply the possible application in tunable capacitor. The high-temperature dielectric relaxation behavior is observed, which is caused by oxygen vacancies with activation energy between 1.059 and 1.318 eV. According to defect symmetry principle, oxygen vacancies in equilibrium ferroelectric state can create defect polarization which provides a restoring force for domain switching. So the pinched loops can be observed. The fast and sufficient motivation of new domain walls to defect dipoles and electric conduction in high-temperature, high-electric-field or low-frequency measurements are deemed to be the main cause for depinning effect. Annealing in oxygen which reduces the concentration of oxygen vacancies and polarizing by applied electric field which can transform the defect polarization to parallel to applied electric field exert enormous impact on pinning effect and result in loops with no or less constriction. Understanding the interaction between oxygen vacancies and ferroelectric domain switching is important for further application.

## Additional Information

**How to cite this article**: Huang, X.-X. *et al*. Dielectric relaxation and pinning phenomenon of (Sr,Pb)TiO_3_ ceramics for dielectric tunable device application. *Sci. Rep.* **6**, 31960; doi: 10.1038/srep31960 (2016).

## References

[b1] Zhang S. . Optoelectronic and Ferroelectric Properties of Cerium-Doped (Na_0.5_Bi_0.5_)(Ti_0.99_Fe_0.01_)O_3_ Nanocrystalline Films on (111) Pt/TiO_2_/SiO_2_/Si: A Composition-Dependent Study. ACS. Appl. Mater. Interfaces. 5, 3191–3198 (2013). 23544846 10.1021/am400196c

[b2] Varghese J., Whatmore R. W. & Holmes J. D. Ferroelectric nanoparticles, wires and tubes: synthesis, characterisation and applications. J. Mater. Chem. C. 1, 2618–2138 (2013).

[b3] Li B. S., Zhu Z. G., Li G. R., Yin Q. R. & Ding A. L. Peculiar Hysteresis Loop of Pb(Mn_1/3_Nb_2/3_)O_3_-Pb(Ti,Zr)O_3_ Ceramics. Jpn. J. Appl. Phys. 43, 1458–1463 (2004).

[b4] Zhang H., Jiang S. L. & Zeng Y. K. B Site Doping Effect on Depinning in Pb(Mn_1/3_Nb_1/3_Sb_1/3_)_*x*_(Zr_0.825_Ti_0.175_)_1–*x*_O_3_ Ferroelectric Ceramics. Appl. Phys. Lett. 93, 192901 (2008).

[b5] Li B. S. . Pinning and Depinning Mechanism of Defect Dipoles in PMnN-PZT Ceramics. J. Phys. D. Appl. Phys. 38, 1107–1111 (2005).

[b6] Yoon S. J., Kang H. W., Kucheiko S. I., Kim H. J. & Jung H. J. Piezoelectric Properties of Pb[Zr_0.45_Ti_0.5-*x*_Lu_*x*_(Mn_1/3_Sb_2/3_)_0.05_]O_3_ Ceramics. J. Am. Ceram. Soc. 81, 2473–2476 (1998).

[b7] Zhang L. X., Chen W. & Ren X. B. Large Recoverable Electrostrain in Mn-doped (Ba,Sr)TiO_3_ Ceramics. Appl. Phys. Lett. 85, 5658 (2004).

[b8] Ren X. B. Large Electric-field-induced Strain in Ferroelectric Crystals by Point-defect-mediated Reversible Domain Switching. Nat. Mater. 3, 91–94 (2004). 14716304 10.1038/nmat1051

[b9] Zhang L. X. & Ren X. B. *In Situ* Observation of Reversible Domain Switching in Aged Mn-doped BaTiO_3_ Single Crystals. Phys. Rev. B. 71, 174108 (2005).

[b10] Liu W. F., Zhang L. X., Chen W., Li S. T. & Ren X. B. Large Digital-characterized Electrostrain in Mn-doped (Pb,Sr)TiO_3_ Electro-shape-memory Ceramics. Appl. Phys. Lett. 99, 092907 (2011).

[b11] Lin D. M., Kwok K. W. & Chan H. L. W. Double Hysteresis Loop in Cu-doped K_0.5_Na_0.5_NbO_3_ Lead-free Piezoelectric Ceramics. Appl. Phys. Lett. 90, 232903 (2007).

[b12] Tan Q., Li J. X. & Viehland D. Role of Lower Valent Substituent-oxygen Vacancy Complexes in Polarization Pinning in Potassium-modified Lead Zirconate Titanate. Appl. Phys. Lett. 75, 418 (1999).

[b13] Giencke J. E., Folkman C. M., Baek S. H. & Eom C. B. Tailoring the domain structure of epitaxial BiFeO_3_ thin films. Curr. Opin. Solid. State. Mater. Sci. 18, 39–45 (2014).

[b14] Pandey S. K., Thakur O. P., Kumar A. & Prakash C. Study of Pinched Loop Characteristics of Lead Zirconate Titanate (65/35). J. Appl. Phys. 100, 014104 (2006).

[b15] Chandrasekaran A., Damjanovic D., Setter N. & Marzari N. Defect ordering and defect-domain-wall interactions in PbTiO_3_: A first-principles study. Phys. Rev. B. 88, 214116 (2013).

[b16] Somiya Y., Bhalla A. S. & Cross L. E. Study of (Sr,Pb)TiO_3_ Ceramics on Dielectric and Physical Properties. Inter. J. Inorg. Mater. 3, 709–714 (2001).

[b17] Zheng Z. . Dipole azimuth dependent permittivity in randomly and (100) oriented (Pb,Sr)TiO_3_ thin films. J. Mater. Chem. 21, 10808–10812 (2011).

[b18] Yang J., Meng X. J., Shen M. R., Sun J. L. & Chu J. H. Effects of Mn doping on dielectric and ferroelectric properties of (Pb,Sr)TiO_3_ films on (111) Pt/Ti/SiO_2_/Si substrates. J. Appl. Phys. 106, 094108 (2009).

[b19] Jiang Y. P., Tang X. G., Zhou Y. C. & Liu Q. X. Pb-Doping Effects on the dielectric and pyroelectric properties of (Sr,Pb)TiO_3_ system. J. Adv. Dielect. 2, 1250005 (2012).

[b20] Morii K., Kawano H., Fujii I., Matsui T. & Nakayama Y. Dielectric relaxation in amorphous thin films of SrTiO_3_ at elevated temperatures. J. Appl. Phys. 78, 1914 (1995).

[b21] Wang X. F. . Oxygen-vacancy-related high-temperature dielectric relaxation in SrTiO_3_ ceramics. J. Appl. Phys. 107, 114101 (2010).

[b22] Wang C. C. . Oxygen-vacancy-related dielectric relaxations in SrTiO_3_ at high temperatures. J. Appl. Phys. 113, 094103 (2013).

[b23] De Souza. R. A. & Maier J. Capacitance of single crystal and low-angle tilt bicrystals of Fe-doped SrTiO_3_. Faraday. Discuss. 134, 235–245 (2007). 17326571 10.1039/b602914k

[b24] Lambeck P. V. & Jonker G. H. The nature of domain stabilization in ferroelectric perovskites. J. Phys. Chem. Solids. 47, 453–461 (1986).

[b25] Hu G. X., Cai X. & Rong Y. H. Fundamentals of Materials Science. ch. 5, pp189–191 (2010).

[b26] Miller R. C. & Savage A. Further Experiments on the Sidewise Motion of 180^o^ Domain Walls in BaTiO_3_. Phys. Rev. 115, 1176 (1959).

[b27] Tagantsev A. K., Sherman V. O., Astafiev K. F., Venkatesh J. & Setter N. Ferroelectric Materials for Microwave Tunable Applications. J. Electroceram. 11, 5–66 (2003).

[b28] Tang X. G., Chew K. H. & Chan H. L. W. Diffuse phase transition and dielectric tunability of Ba(Zr_y_Ti_1-y_)O_3_ relaxor ferroelectric ceramics. Acta. Mater. 52, 5177–5183 (2004).

[b29] Li C. H., Zhang X. Q., Cheng Z. H. & Sun Y. Room temperature giant dielectric tunability effect in bulk LuFe_2_O_4_. Appl. Phys. Lett. 92, 182903 (2008).

[b30] Zhang T. F. . Oxygen-vacancy-related relaxation and conduction behavior in (Pb_1-*x*_Ba_*x*_)(Zr_0.95_Ti_0.05_)O_3_ ceramics. AIP. Adv. 4, 107141 (2014).

[b31] Elissalde C. & Ravez J. Ferroelectric ceramics: defects and dielectric relaxations. J. Mater. Chem. 11, 1957–1967 (2001).

[b32] Zhang T. F., Tang X. G., Liu Q. X., Jiang Y. P. & Huang X. X. Oxygen-Vacancy-Related High Temperature Dielectric Relaxation in (Pb_1-*x*_Ba_*x*_)ZrO_3_ Ceramics. J. Am. Ceram. Soc. 98, 551–558 (2015).

[b33] Zhang T. F. . Energy-storage properties and high-temperature dielectric relaxation behaviors of relaxor ferroelectric Pb(Mg_1/3_Nb_2/3_)O_3_-PbTiO_3_ ceramics. J. Phys. D: Appl. Phys. 49, 095302 (2016).

[b34] Zhang T. F. . High-Temperature Dielectric Relaxation Behaviors of Relaxer-Like PbZrO_3_-SrTiO_3_ Ceramics for Energy-Storage Applications. Energy. Technol. 4, 633–640 (2016)

[b35] Cheng B. L., Button T. W. & Gabbay M. Oxygen Vacancy Relaxation and Domain Wall Hysteresis Motion in Cobalt-Doped Barium Titanate Ceramics. J. Am. Ceram. Soc. 88, 907–911 (2005).

[b36] Scott J. F. & Dawber M. Oxygen-vacancy Ordering as a Fatigue Mechanism in Perovskite Ferroelectrics. Appl. Phys. Lett. 76, 3800 (2000).

[b37] Warren W. L., Vanheusden K., Dimos D., Pike G. E. & Tuttle B. A. Oxygen Vacancy Motion in Perovskite Oxides. J. Am. Ceram. Soc. 79, 536–538 (1996).

[b38] Zhang L. X., Erdem E., Ren X. B. & Eichel R. A. Reorientation of (Mn″_Ti_-V¨_O_)^×^ Defect Dipoles in Acceptor-modified BaTiO_3_ Single Crystal: An Electron Paramagnetic Resonance Study. Appl. Phys. Lett. 93, 202901 (2008).

[b39] Morozov M. I. & Damjanovic D. Charge migration in Pb(Zr,Ti)O_3_ ceramics and its relation to ageing, hardening, and softening. J. Appl. Phys. 107, 034106 (2010).

[b40] Lee J. . Effect of oxygen vacancy on the structural and electronic characteristics of crystalline Zn_2_SnO_4_. J. Mater. Chem. C. 2, 8381–8387 (2014).

[b41] Salari M., Konstantinov K. & Liu H. K. Enhancement of the capacitance in TiO_2_ nanotubes through controlled introduction of oxygen vacancies. J. Mater. Chem. 21, 5128–5133 (2011).

[b42] Yang C., Zhu Q., Lei T., Li H. & Xie C. The coupled effect of oxygen vacancies and Pt on the photoelectric response of tungsten trioxide films. J. Mater. Chem. C. 2, 9467–9477 (2014).

